# Immobilization of Cr(VI) in Soil Using a Montmorillonite-Supported Carboxymethyl Cellulose-Stabilized Iron Sulfide Composite: Effectiveness and Biotoxicity Assessment

**DOI:** 10.3390/ijerph17176087

**Published:** 2020-08-21

**Authors:** Dading Zhang, Yanqiu Xu, Xiaofei Li, Zhenhai Liu, Lina Wang, Chaojun Lu, Xuwen He, Yan Ma, Dexun Zou

**Affiliations:** 1 School of Chemical and Environmental Engineering, China University of Mining and Technology (Beijing), Beijing 100083, China; tmaczdd02@163.com (D.Z.); xuyq15871041327@163.com (Y.X.); m18850341814@163.com (L.W.); 108137@cumtb.edu.cn (X.H.); 2 College of Chemical Engineering, Beijing University of Chemical Technology, Beijing 100029, China; lixiaofei203@126.com; 3 College of Environmental Science and Engineering, Nankai University, Tianjin 300350, China; hiram0823@163.com; 4 Chinese Research Academy of Environmental Sciences, Beijing 100012, China; lucj@craes.org.cn

**Keywords:** potential toxic metal contamination, CMC@MMT-FeS, soil remediation, BCR test, toxicity characteristic leaching procedure, biotoxicity assessment

## Abstract

A novel composite of montmorillonite-supported carboxymethyl cellulose-stabilized nanoscale iron sulfide (CMC@MMT-FeS), prepared using the co-precipitation method, was applied to remediate hexavalent chromium (Cr(VI))-contaminated soil. Cr(VI)-removal capacity increased with increasing FeS-particle loading. We tested the efficacy of CMC@MMT-FeS at three concentrations of FeS: 0.2, 0.5, and 1 mmol/g, hereafter referred to as 0.2 CMC@MMT-FeS, 0.5 CMC@MMT-FeS, and 1.0 CMC@MMT-FeS, respectively. The soil Cr(VI) concentration decreased by 90.7% (from an initial concentration of 424.6 to 39.4 mg/kg) after 30 days, following addition of 5% (composite–soil mass proportion) 1.0 CMC@MMT-FeS. When 2% 0.5 CMC@MMT-FeS was added to Cr(VI)-contaminated soil, the Cr(VI) removal efficiency, as measured in the leaching solution using the toxicity characteristic leaching procedure, was 90.3%, meeting the environmental protection standard for hazardous waste (5 mg/kg). The European Community Bureau of Reference (BCR) test confirmed that the main Cr fractions in the soil samples changed from acid-exchangeable fractions to oxidable fractions and residual fractions after 30 days of soil remediation by the composite. Moreover, the main complex formed during remediation was Fe(III)–Cr(III), based on BCR and X-ray photoelectron spectroscopy analyses. Biotoxicity of the remediated soils, using *Vicia faba* and *Eisenia foetida*, was analyzed and evaluated. Our results indicate that CMC@MMT-FeS effectively immobilizes Cr(VI), with widespread potential application in Cr(VI)-contaminated soil remediation.

## 1. Introduction

Potential toxic metal contamination is a global concern, because toxic metals can bioaccumulate in the food chain, causing high levels of toxicity in the human body [1,2,3]. Chromium (Cr), a potential toxic metal, is often released into water or soil from chromate manufacturing, textile dyeing, tanneries, and metal electroplating, due to poor waste management [4,5]. Cr(III) and Cr(VI) are two common species of Cr in the environment. Cr(III)—which comes from insoluble substances such as Cr(OH)_3_ and Cr_2_O_3_—has a relatively low biotoxicity and is more stable; Cr(VI)—which comes from compounds such as CrO_4_^2−^, HCrO_4_^−^, and Cr_2_O_7_^2−^—is easier to mobilize than Cr(III), particularly in soil [6,7,8]. The biological toxicity of Cr(VI) is over 100 times that of Cr(III) [9]. Thus, Cr(VI) is regarded as the most bio-toxic form of Cr; its concentration in water and soil should be kept low, owing to its high toxicity, carcinogenicity, and potential for bioaccumulation [10,11].

Cr(VI) can be converted to Cr(III) in the presence of reductants, such as ferrous iron, manganous ion, organic phenols, and a reducing microorganism [12,13]. Cr(III) can be oxidized to Cr(VI) through interaction with manganese dioxide, and the conversion is substantially influenced by the equilibrium between the dissolved and solid phases of Cr(III); furthermore, the kinetics are slow [14]. When Cr(VI) is converted into Cr(III), various precipitant compounds are formed, such as Cr(OH)_x_, Cr_2_O_3_, and Fe(III)–Cr(III) hydroxides, which reduce its risk to humans [15,16].Therefore, converting easily mobile and highly toxic Cr(VI) compounds into stable and low toxicity Cr(III) compounds is an effective remediation strategy to alleviate the threat of Cr(VI) to ecosystems.

As urbanization increases, so do the number of Cr(VI)-contaminated sites in need of remediation, and these sites can then be used for residential or commercial purposes [17]. In recent years, methods such as bioremediation, chemical reduction, and immobilization have been used to remove Cr(VI) from water and soil [18,19,20]. Chemical reduction is widely used owing to its high efficiency and low cost [21]. Inorganic or organic electron donors can reduce Cr(VI) to Cr(III) and form insoluble Cr(III) hydroxides in the reduction process [22,23,24]. Iron minerals, such as goethite, hematite, mackinawite, and pyrite, in soil and subsurface sediment have been shown to reduce Cr(VI) to Cr(III) [25,26,27,28]. However, the reduction rate is slow, and it depends on the iron content in minerals [29]. Ferrous compounds, especially ferrous sulfate (FeSO_4_), have been widely studied and used in the remediation of Cr(VI)-contaminated sites [30]. However, this approach can cause various problems when the pure ferrous reagent is added to a Cr(VI)-contaminated solution or soil, including excess production of reagents in soil, inadequate reduction of Cr(VI), and an overload of anions in soil particles. Hence, many alternative species of iron materials have been developed for the remediation of Cr(VI)-contaminated water or soil. Owing to the highly active nature of nanomaterials, the removal of Cr(VI) by nanoscale zero-valent iron (nZVI) and biochar-, rock wool-, and graphite-loaded nZVI has been widely studied [31,32,33,34]. However, the use of nZVI has many drawbacks, including high costs and strict reductive conditions, limiting its application.

Recent studies have shown that iron sulfide (FeS) is highly efficient in immobilizing Cr(VI) in water or soil because it can provide Fe(II) and S(-II) species that reduce Cr(VI) to Cr(III). Compared with iron sulfide-containing minerals, FeS particles are small with a large specific surface area, which contributes to their higher reactivity [35]. They can absorb Cr(VI) via surface reactions [36]. However, FeS particles tend to aggregate, impeding their effectiveness [37]. Therefore, FeS-modified materials are being studied: for example, chitosan-stabilized FeS effectively remediated Cr(VI)-contaminated aqueous samples [38], and carboxymethyl cellulose (CMC)-stabilized FeS remediated Cr(VI)-contaminated water and soil [39,40,41]. Here, we present a novel composite comprising CMC-stabilized FeS on montmorillonite (MMT), preventing the aggregation of the FeS particles and enhancing their dispersal and utilization in soils. CMC, as a common anionic high-polymer cellulose, can interact with multivalent cations owing to its numerous carboxyl and hydroxyl groups. It is usually applied as a stabilizer in suspension owing to its electrostatic repulsion and space-resistance effects [42,43,44]. MMT, as a typical 2:1 phyllosilicate resource, is abundant, inexpensive, and environmentally friendly, and has been frequently applied as an adsorbent or supporting material in environmental remediation [45,46]. Its large surface area contributes to the dispersion of FeS particles. Acid modification can remove impurities from the MMT, and further enhance the mineral pore spaces and support more target substance [47].

This study used an MMT-supported and CMC-stabilized FeS composite (CMC@MMT-FeS) to remediate Cr(VI) contamination in soil and aqueous samples. We investigated the following aspects of this novel composite: (1) its composition and surface properties; (2) how effectively it remediates Cr(VI) contamination, compared with an FeSO_4_ reagent; (3) the Cr fractions in the soil before and after remediation and the mechanisms of Cr(VI) immobilization; and (4) the biotoxicity of soils before and after remediation. Our objective is to provide a reference for the use of CMC@MMT-FeS to remediate Cr(VI)-contaminated environments.

## 2. Materials and Methods

### 2.1. Chemicals and Materials

Ultrafine MMT powder was purchased from Lingshou Mineral Powder Factory (Shijiazhuang, China). The analytical-grade chemicals, including sodium sulfide nonahydrate (Na_2_S·9H_2_O), ferrous sulfate heptahydrate (FeSO_4_·7H_2_O), potassium chromate (K_2_CrO_4_), sodium nitrate (NaNO_3_), acetic acid (C_2_H_4_O_2_), hydrochloric acid (HCl), sodium hydroxide (NaOH), and ethanol were purchased from the Beijing Chemical Regent Factory (Beijing, China). CMC was purchased from Anpel Laboratory Technologies (Shanghai, China). All solutions were prepared with pure water of >18 MΩ cm^−1^.

The soil samples were collected from an electroplating workshop of a factory in Hefei, China, and the Cr(VI) content in the soil was 24.5 ± 2 mg/kg, which was below the risk intervention value [48]. Stones and plant roots were removed from the soil samples, which were air-dried, ground, and then sieved through a 2 mm standard mesh. The physicochemical properties of the soil samples are shown in Table S1 (Supplementary Materials). Cr(VI)-contaminated soil was prepared as follows: 1 L of K_2_CrO_4_ solution at a concentration of 1.865 g/L was slowly mixed with 1 kg of soil, and then homogenized by vigorous stirring. The pretreated soil was then air-dried under natural conditions and aged for 60 d. The aged soil samples were pulverized into fine particles and passed through a 2 mm standard sieve. The pulverized soil was stored in a zip-lock bag in dark conditions until further analysis. The Cr(VI) concentration of the prepared contaminated soil samples was 424.6 ± 17 mg/kg.

### 2.2. Preparation and Characterization of CMC@MMT-FeS

The composite CMC@MMT-FeS sample was synthesized via a chemical co-precipitation method, as previously reported [5]. First, 9.54 g of purified MMT (Supplementary Materials Section 1) was transferred into a 2 L anaerobic Erlenmeyer flask containing 1 L of pure water; high purity nitrogen gas was added to remove the dissolved oxygen. After 1 h, 20 mL of freshly prepared 2.5 mmol/L FeSO_4_ solution was added to this mixture, which was magnetically stirred for 30 min while nitrogen was continuously purged into the flask, resulting in the formation of MMT-Fe^2+^. Subsequently, 3.82 mL of CMC solution (0.5%, *w*/*w*) was added, resulting in the formation of CMC@MMT-Fe^2+^ complexes. Finally, 10 mL of 6 mmol/L Na_2_S solution was added drop by drop to the mixture with continuous magnetic stirring. The S^2−^ combined with the CMC@MMT-Fe^2+^ to form the CMC@MMT-FeS composite containing 0.5 mmol/g FeS. The chemical reaction is shown in Equations (1)–(3).

(1) $$\mathrm{MMT}\;+\;\mathrm{Fe}{\left(H_{2}O\right)}_{6}{}^{2+}\;\to \;\mathrm{MMT}-\mathrm{Fe}{\left(H_{2}O\right)}_{6}{}^{2+}$$

(2) $$\mathrm{MMT}-\mathrm{Fe}{\left(H_{2}O\right)}_{6}{}^{2+}+\;\mathrm{CMC}\;\to \;\mathrm{CMC}@\mathrm{MMT}-\mathrm{Fe}{\left(H_{2}O\right)}_{6}{}^{2+}$$

(3) $$\mathrm{CMC}@\mathrm{MMT}-\mathrm{Fe}{\left(H_{2}O\right)}_{6}{}^{2+}+{\;S}^{2-}\;\to \;\mathrm{CMC}@\mathrm{MMT}-\mathrm{FeS}$$

The final composite, containing FeS at 0.2, 0.5, and 1 mmol/g (hereafter 0.2 CMC@MMT-FeS, 0.5 CMC@MMT-FeS, and 1.0 CMC@MMT-FeS, respectively), was prepared using the above procedure. The precipitant was filtered and vacuum freeze-dried for subsequent use.

The crystalline structures of MMT and CMC@MMT-FeS particles were visualized using X-ray diffraction (XRD, Shimadzu Corporation, Japan) with Cu-K_α_ radiation (λ = 0.15418 nm). The surface chemicals were measured using Fourier transform infrared spectroscopy (FTIR, MODEL 205, Nicolet Corporation, USA) equipped with a deuterated triglycine sulfate detector. The specific surface areas and internal microporous properties of the MMT and composite were analyzed using the Brunauer–Emmett–Teller (BET) method via N_2_ adsorption–desorption at 77 K, on an ASAP 2460 Surface Area and Porosity Analyzer (Micromeritics, Norcross, GA, USA).

### 2.3. Remediation of Cr(VI)-Contaminated Soil

Fourteen Cr-contaminated soil samples (100 g) (prepared as per Section 2.1) were weighed into 750 mL polypropylene plastic boxes. To explore the process of Cr(VI) immobilization in soil using the composite materials, the Cr(VI) concentration and oxidation–reduction potential (ORP) in the soil samples were measured at specific times (1, 2, 3, 5, 7, 10, 15, and 30 d) in three treatments, comprising the addition of 0.5 CMC@MMT-FeS, 1.0 CMC@MMT-FeS (5% composite–soil mass proportions), and 2.5 mmol FeSO_4_ reagent. To assess the optimum reaction dosage and Cr(VI) immobilization effect, 1%, 2, 3, 5, and 10% doses (composite–soil mass proportions) of the 0.5 CMC@MMT-FeS were added to the contaminated soil for remediation. Additionally, 0.5, 1.0, 1.5, 2.5, and 5.0 mmol of the FeSO_4_ reagent, providing the same amount of Fe(II) as the 1, 2, 3, 5, and 10% doses of the 0.5 CMC@MMT-FeS, were added. A blank control group (CK, containing no immobilization complex or reagent) was also prepared. The sample mixtures were stirred well, and then the container was sealed and incubated for 60 d at room temperature (23 ± 2 °C). During the incubated period, the soil moisture content at 50% was maintained by weighing method. The effectiveness of Cr(VI) immobilization in the soil was estimated by measuring the concentrations of Cr(VI) in remediated soils. The Cr(VI) immobilization efficiency was calculated as per Equation (4):

(4) $$\mathrm{Immobilization}\;\mathrm{efficiency}\;\left(\%\right)=\;\left(1\;-C_{t}/C_{0}\right)\;\times \;100$$

where C_t_ and C_0_ are the Cr(VI) concentrations (mg/kg) in the contaminated soil at time t and initially, respectively. All experiments were conducted in an anaerobic environment, in duplicate.

### 2.4. Evaluation of Cr(VI) Stability in the Soil after Remediation

Toxicity characteristic leaching procedure (TCLP) tests were performed to evaluate the potential for metal immobilization in the untreated soil, and in soils remediated with the composite and FeSO_4_. The TCLP experiments followed the Environmental Protection Industry Standard of China (China, HJ/T 300-2007) [49]. In short, 2 g of soil samples before and after remediation were transferred into 50 mL centrifuge tubes, and then 40 mL of extraction solution (5.7 mL 99–100%acetic acid and 64.3 mL 1 M NaOH in 1 L pure water, pH 4.93 ± 0.05) was added to obtain a liquid-to-solid ratio of 20:1. The samples were shaken rotationally for 18 h at room temperature (23 ± 2 °C). The supernatants containing the Cr(VI) were then passed through a 0.8 µm micro-filter for analysis.

The three-step sequential procedure proposed by the European Community Bureau of Reference (BCR) was used to analyze heavy metals fractions in the soil [50]. Fractions of Cr in the soil before and after remediation were extracted using a modified three-step sequential procedure [51], for extraction of (1) the acid-exchangeable fraction, (2) the reducible fraction, and (3) the oxidizable fraction, followed by extraction of the residual fraction (Supplementary Materials Section 2, Table S2). The supernatant liquids were kept at 4 °C for subsequent measurements.

### 2.5. Biotoxicity Assessment of Cr(VI)-Contaminated Soils after Remediation

To explore the effect of CMC@MMT-FeS on the biotoxicity of Cr(VI)-contaminated soil, *Vicia faba* and *Eisenia foetida* were used for biotoxicity testing, using the contaminated soil before and after remediation [52,53].

The *V. faba* micronucleus test was conducted according to Kanaya et al. [54,55,56]. The secondary roots of *V. faba* (1–2 cm long, Supplementary Materials Section 3) were suitable for the micronucleus test. The secondary roots were immersed in a Petri dish of soil extract (Supplementary Materials Section 4) for 24, 48, or 72 h at room temperature (23 ± 2 °C). The roots from at least two seedlings were tested in each extract, and five root tips were randomly selected from each seedling. The secondary roots were then placed into Carnoy’s solution (acetic acid: ethanol 1:3, *v*/*v*) and fixed overnight at 4 °C. Next, they were cleaned with deionized water and transferred to 70% ethanol for storage. They were hydrolyzed in 1 M HCl for 15 min at 60 °C. Thereafter, 1 mm of root-tip meristem was placed on a glass slide with 1% aceto-orcein reagent (1 g orcein, 45 mL acetic acid, 55 mL deionized water) for staining for 15 min. Then, roots were washed, pressed, and microscopically examined. At least 1000 cells were scored for each root tip. The pollution index (PI), an important index for evaluating the degree of environmental toxicity in living organisms, was calculated using Equation (5):

(5) $$\mathrm{PI}\;=\;MCN_{\mathrm{remediation}}/MCN_{CK}$$

where *MCN_remediation_* and *MCN_CK_* are the *V. faba* micronucleus rates in the remediation and CK groups, respectively.

According to Sivakumar and Subbhuraam (2005), the median lethal concentration of Cr(VI) for *E. foetida* is about 220 mg/kg [57]. Background soil was added to the experimental soil samples to achieve the proper dilution. The Cr(VI) concentrations in the original and diluted soil after remediation are shown in Table S3. Samples of 300 g diluted soil and 10 randomly selected *E. foetida*, which had been incubated in the laboratory environment for two weeks, were placed in a 1 L beaker, which was placed in dark conditions at room temperature (23 ± 2 °C). The daily mortality of the earthworms was recorded. One or two *E. foetida* were taken for intestinal cleaning, and weighed at 1, 3, 7 and 14 d. They were then cut into pieces and homogenized in buffer solution at a solid–liquid mass ratio of 1:9. The tissue solution was transferred and centrifuged at 3000 rpm for 10 min. The superoxide dismutase (SOD) and peroxidase (POD) activity levels in the supernatants were measured using commercial kits (A001-3-2 and A084-1-1, Jiancheng Bioengineering Institute, Nanjing, China).

### 2.6. Analysis Methods

Cr(VI) in soils was extracted using a heater with MgCl_2_ and phosphate buffer (pH = 7) in alkaline medium (Na_2_CO_3_/NaOH solution), and its concentration was measured using flame atomic absorption spectrophotometry (China, HJ/T 687-2014) [58]. Cr(VI) concentrations in the solutions were determined using a UV2200 spectrophotometer (Shanghai Sunny Hengping Scientific Instrument Co., Ltd., Shanghai, China) following the diphenyl-carbazide spectrophotometric method (China, GB 7467-87) [59]. The soil redox potential was determined according to the China national standard SL 94-1994, using an SX731 portable redox potential tester (Sanxin, Shanghai, China). In addition, the surface elements of the soil before and after remediation were investigated via X-ray photoelectron spectroscopy (XPS, Escalab 250, Thermo Fisher, USA) with a monochromatic Al Kα source.

## 3. Results and Discussion

### 3.1. Characterization of Materials

**Figure 1:**
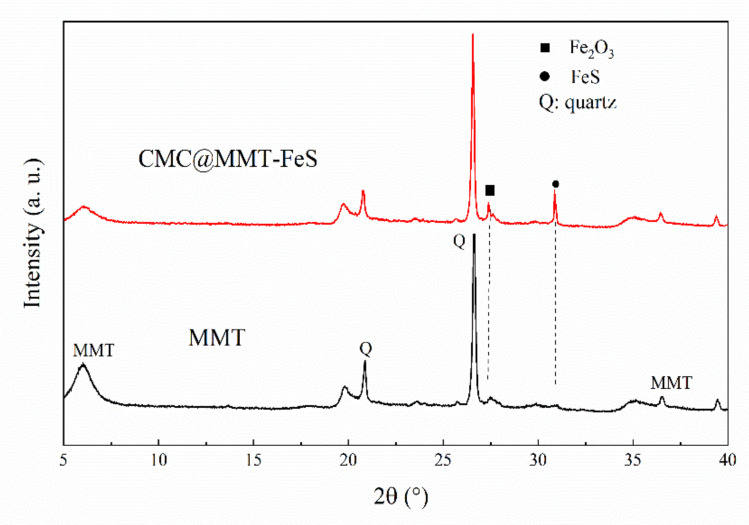
X-ray diffraction (XRD) pattern analysis of montmorillonite (MMT) and carboxymethyl cellulose-stabilized nanoscale iron sulfide (CMC@MMT-FeS).

Modified MMT and raw materials were examined using the XRD technique; patterns with a diffraction angle of 2θ ranging from 5° to 40° are shown in Figure 1. The composite diffraction pattern had two new peaks, at 27.5 Å and 30.8 Å, which were attributed to the presence of Fe_2_O_3_ and FeS, respectively [60,61]. This indicates that nano-FeS was successfully loaded on the surface layers of the MMT. Additionally, Fe_2_O_3_ may be a product of the oxidation of Fe(OH)_2_ and Fe(OH)_3_; it is also possible that FeS was oxidized and partly deactivated. By estimating the basic spacing of D_001_, we found that the interlayer distance of the modified MMT did not expand, indicating that nano-FeS had not formed in the clay interlayer space. This provides further evidence that the FeS nanoparticles were mainly loaded on the surfaces of the MMT.

**Figure 2:**
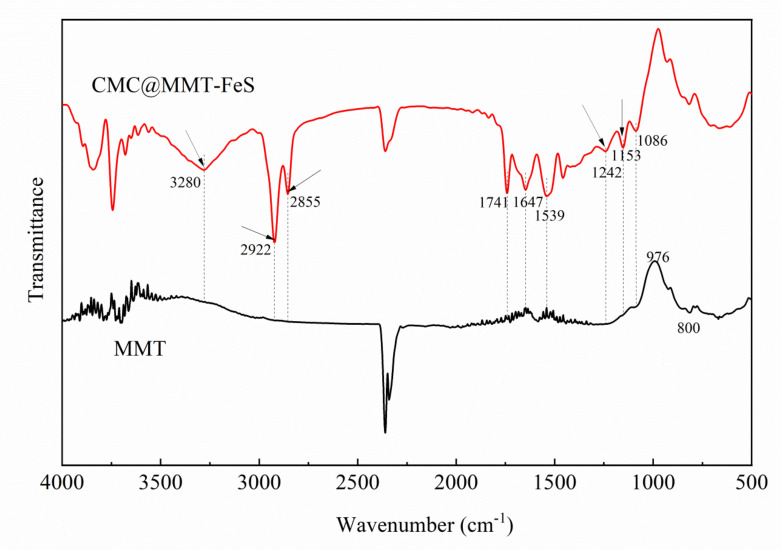
Fourier transform infrared spectral pattern analyses of montmorillonite (MMT) and CMC@MMT-FeS.

The characteristic stretching frequencies of the MMT and modified materials were compared using the FTIR spectra (Figure 2). The characteristic peaks were mainly distributed in the 500–4000 cm^−1^ range. The changes in transmittance at 976 and 800 cm^−1^ were related to the Si–O bond bending vibration and Mg–OH stretching vibration. The CMC@MMT-FeS spectrum had new peaks at 3280, 2922, 2855, 1242, and 1153 cm^−1^. The 3280 cm^−1^ peak was attributed to the stretching of N–H bonds [62]. The 2922 and 2855 cm^−1^ peaks were attributed to the asymmetric and symmetric stretching vibration of the C–H bonds in the -CH_2_ group [63], whereas the 1242 and 1153 cm^−1^ peaks were attributed to C–O–C stretching vibration [64]. There were relatively more intense stretching bands at 1741 and 1647 cm^−1^, which can be attributed to the C=O stretching vibration, and to the C–O or N–H bending vibration, respectively. The 1539 cm^−1^ peak was attributed to carboxylate absorption. These results reveal that nano-FeS particles were attached to CMC via the carboxylate and hydroxyl groups, and that functional groups such as C=O, C–O–C, and N–H may play important roles in the process of loading CMC and nano-FeS particles onto the surface of the MMT.

The specific surface parameters of the MMT and the CMC@MMT-FeS were studied using Brunauer–Emmett–Teller (BET) analysis. The specific surface area of the MMT increased from 84.24 to 88.98 m^2^/g, but the pore volume decreased slightly from 0.110 to 0.107 cm^3^/g after modification. The augmentation of the specific surface area was a result of FeS particles being loaded onto the surface of MMT and increasing the MMT surface roughness. Some FeS particles were generated by co-precipitation in the pores, which partly blocked the pores of MMT, thereby reducing the unit pore volume. The BET analysis results show that, although the FeS particles in the composite were deposited mainly on the surface of the material, they also formed in the pores of the MMT.

### 3.2. Effect of Cr(VI) Immobilization in Soil by CMC@MMT-FeS

The removal efficacy of the different prepared composite on Cr(VI) in aqueous solution was assessed during preliminary experiments (Supplementary Materials Section 5): in solution, the Cr(VI) removal capacities of the three types of composite, ranked in order of capacity, were as follows: 1.0 CMC@MMT-FeS > 0.5 CMC@MMT-FeS > 0.2 CMC@MMT-FeS (Figure S1). In aqueous solution, the Cr(VI)-removal capacity of the composite was positively associated with FeS loading. Additionally, pseudo-first-order and pseudo-second-order kinetic models were used to fit the amount of Cr(VI) removed by the modified material in aqueous solution; the removal process followed the pseudo-second-order kinetic model more closely (Table S4). This is consistent with the results of Lyu et al., who reported that Cr(VI) removal with biochar-supported nanoscale iron sulfide composite fitted a pseudo-second-order kinetic model better, indicating that the rate-limiting step for Cr(VI) removal was chemisorption [5].

**Figure 3:**
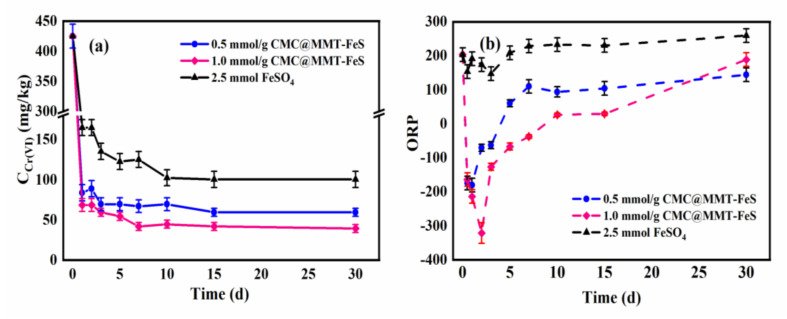
(**a**) The concentration of hexavalent chromium (Cr(VI)) and (**b**) the oxidation–reduction potential (ORP) of soil samples after amendments over 30 days (added composite–soil mass proportion: 5%).

The Cr(VI) content in soil samples during aging, after 5% of the 0.5 or 1.0 CMC@MMT-FeS, or 2.5 mmol FeSO_4_ were added to remediate the contaminated soil, is shown in Figure 3a: the immobilization of Cr(VI) in contaminated soil occurred mainly in the first 15 days, and the content of Cr(VI) in soil tended to stabilize after 15 days of remediation. The concentration of Cr(VI) in the soil showed a sharp decrease on the first day, and then a gradual decrease, becoming more stable. After aging for 30 days, the concentration of Cr(VI) in soil samples treated with 0.5 CMC@MMT-FeS, 1.0 CMC@MMT-FeS, and FeSO_4_ decreased by 86, 90.7, and 76.3%, respectively. Compared to the FeSO_4_ reagent, the composite containing the same molar content of Fe^2+^ immobilized Cr(VI) from the soil 9.6% more efficiently. Comparing the FeS loading of the composite, 1.0 CMC@MMT-FeS immobilized Cr(VI) about 4.8% more efficiently than 0.5 CMC@MMT-FeS, when the proportion of composite added was 5%.

Figure 3b shows the oxidation–reduction potential (ORP) of soil samples during the remediation process using the three treatments: after addition of FeSO_4_, the ORP of the soil environment decreased for the first three days, then gradually increased, reaching about 260 mV after 30 days. After addition of 0.5 CMC@MMT-FeS, the soil ORP decreased to about −180 mV on the first day, and then increased gradually, reaching 60 mV on the fifth day, and increasing to 144.5 mV after 30 d. After addition of 1.0 CMC@MMT-FeS, the soil ORP decreased to a minimum value of −321 mV after two days, and gradually increased to 26 mV after 10 days, and to 188 mV after 30 days. The ORP data reveal that the contaminated soil was oxidizable, and that the ORP of the soil went through two stages after the amendments: a sudden drop followed by a slow rise. Comparing the three remediation materials, the modified composite greatly reduced soil ORP and improved the redox capacity. Additionally, increases in FeS loading on the MMT surface improved the reduction ability of the composite.

The process of Cr(VI) reduction in the soil by the composites was similar to that of Cr(VI) alteration after reduction by nano-FeS particles, described by Li et al. [37], that is, decreasing sharply in 3 d and then reaching equilibrium slowly. The Cr(VI) immobilization efficiency of the composite was higher than that of FeSO_4_, and there are four potential explanations: (1) the Fe(II) in the loaded FeS was more stable and presented stronger reducibility; (2) in addition to Fe(II), the S(-II) in the modified composite might reduce the Cr(VI) in the soil under acidic conditions; (3) the surface properties of the MMT were improved after FeS loading; and (4) the adsorption of modified mineral materials contributed to Cr(VI) immobilization in soil.

### 3.3. Cr(VI) Concentration in Soil Samples before and after Remediation

**Figure 4:**
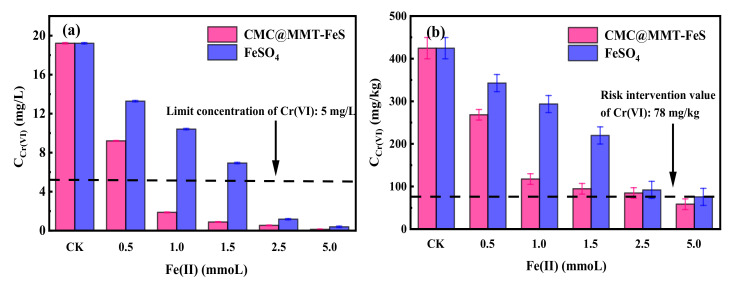
Concentration of Cr(VI) (**a**) in leaching by toxicity characteristic leaching procedure (TCLP) tests, and (**b**) in soil samples before and after soil remediation using 0.5 CMC@MMT-FeS (i.e., composite containing FeS at 0.5 mmol/g) and the FeSO_4_ reagent. CK: control check.

Soil Cr(VI) before and after remediation with the 0.5 CMC@MMT-FeS and FeSO_4_ reagents was tested using TCLP, to study their effects on Cr(VI) immobilization. The TCLP leaching concentration of Cr(VI) in the untreated soil was 19.2 mg/L after 60 days (Figure 4a). The addition of CMC@MMT-FeS at mass proportions of 1, 2, 3, 5, and 10% reduced the leaching concentration of Cr(VI) to 9.2, 1.86, 0.88, 0.53, and 0.12 mg/L, respectively. This demonstrated that the composite reduced Cr(VI) in soil, and thus reduced the leachability and bioavailability of Cr(VI). After the addition of 2% CMC@MMT-FeS, the Cr(VI) concentration in the TCLP extracts was substantially below the standard limit, and the remediated soil could be regarded as non-hazardous waste (China, GB 5085.3-2007) [65]. In contrast, FeSO_4_ reagent remediation (at 0.5, 1.0, 1.5, 2.5, and 5.0 mmol) reduced the leaching concentration of Cr(VI) 13.28, 10.41, 6.93, 1.17, and 0.39 mg/L, respectively. This suggests that CMC@MMT-FeS performed better than FeSO_4_ in immobilizing Cr(VI) under the same Fe(II) conditions. Adding composite at a composite–soil mass proportion of 2% (containing 1.0 mmol Fe(II)) achieved the environmental protection standards for hazardous wastes, whereas 2.5 mmol FeSO_4_ reagent was needed to meet this standard. About twice as much FeSO_4_ reagent as composite was needed to achieve the same level of remediation.

Figure 4b displays the Cr(VI) concentration in soil remediated by 0.5 CMC@MMT-FeS and FeSO_4_ reagents: the composite immobilized Cr(VI) more effectively than FeSO_4_ under the same amount of added Fe(II). In particular, at Fe(II) concentrations of about 1.0–1.5 mmol, the composite immobilized more than twice as much Cr(VI) than FeSO_4_ did. The Cr(VI) concentration in soil amended by CMC@MMT-FeS and FeSO_4_ reagents (containing 5.0 mmol Fe(II)) decreased from 424.6 mg/kg to 58.3 and 75.4 mg/kg, respectively. This suggests that adding 0.5 CMC@MMT-FeS at a composite–soil mass proportion of 10% to the contaminated soil was able to meet the risk-intervention requirement value for second-class construction land (China, GB 36600-2018) [48]. Additionally, the total Cr concentration in the soil before and after remediation is presented in Figure S2, and the results indicated that the total Cr concentration in the soil decreased by 7.1–10.6% after the addition of composite materials at different proportions. In terms of leachability of Cr(VI) and the concentration of Cr(VI) and total Cr in soil, the novel CMC@MMT-FeS showed better performance in terms of immobilizing Cr(VI) in soil than FeSO_4_ reagents.

### 3.4. BCR Tests of Soil Samples before and after Remediation

**Figure 5:**
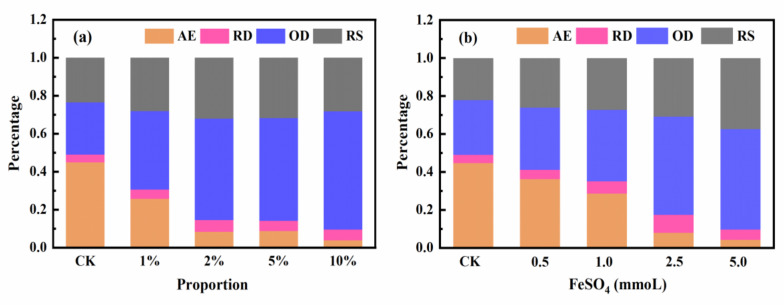
Proportion of Cr in each fraction of the soil samples before and after remediation with (**a**) 0.5 CMC@MMT-FeS; and (**b**) FeSO_4_ reagent. Fractions were measured using the modified BCR (European Community Bureau of Reference) method (AE, acid-exchangeable fraction; RD, reducible fraction; OD, oxidable fraction; RS, residual fraction). CK: control check.

The fractions of potential toxic metals in the soil influence their bioavailability and stability. The bioavailability of heavy metal fractions, ranked from highest to lowest, is as follows: acid-exchangeable (AE) fraction > reducible (RD) fraction > oxidizable (OD) fraction > residual (RS) fraction. The soil samples before and after the amendments were tested using the improved BCR procedure Figure 5: the abundances of the Cr fractions in the soil samples were ranked as follows: AE > RD > OD > RS. Figure 5a shows the changes in the fractions of Cr in untreated and treated soils after the addition of 0.5 CMC@MMT-FeS at composite–soil mass proportions of 0, 1, 3, 5, and 10%. The primary Cr fractions in the untreated soil were AE (44.7%), RD (4.3%), OD (28.1%), and RS (22.9%). As the proportion of composite added increased, the AE fraction decreased from 44.7 to 25.7, 8.2, 8.7, and 3.8%, respectively, while the OD fraction increased from 28.1 to 48.3, 53.5, 54.1, and 62.3%, respectively. The addition of composite to the soil caused the RS fraction to increase by 5.2–9.1%. With the increase in the dosage of FeSO_4_ (0.5, 1.5, 2.5, or 5.0 mmol), the AE fraction decreased from 44.7 to 36.3, 28.6, 7.8, and 4.1%, respectively, while the OD fraction increased from 28.1 to 32.8, 37.7, 51.7, and 53%, respectively (Figure 5b). The RS fraction in the FeSO_4_-remediated soil samples increased by 3.2–14.5%. No apparent changes in the RD fraction were detected in soil treated with composite or FeSO_4_ reagent. This suggests that the addition of composite caused the soil Cr fractions to change from AE to OD and RS, predominantly OD, accounting for the reduced leachability and enhanced stability of Cr(VI) in soil after treatment, thus reducing the toxicity of Cr(VI). According to the results of the present and previous studies, Cr_2_O_3_, Cr(OH)_3_, and Cr(III)-Fe(III) oxides or hydroxides may have been formed during the remediation process [5,40]. Besides, the slight decrease in total Cr concentration in the remediated soil could be ascribed to the increase in the RS fraction of Cr.

### 3.5. The Mechanisms of Cr(VI) Immobilization by CMC@MMT-FeS

**Figure 6:**
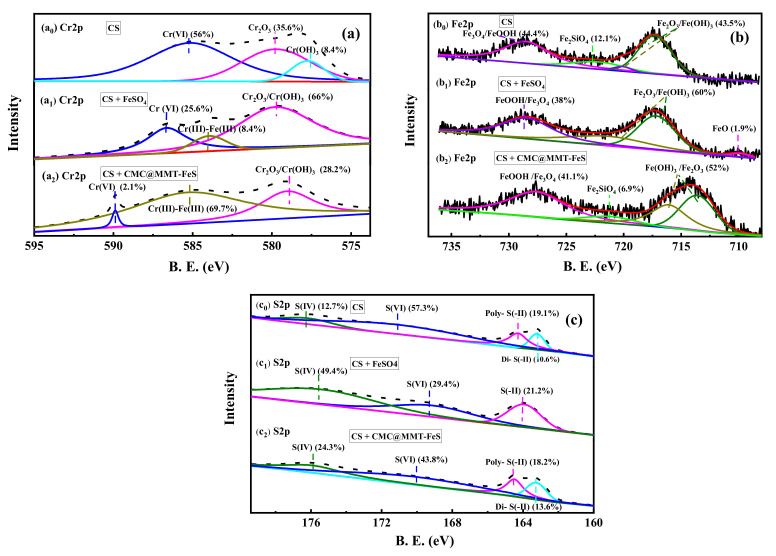
Chemical composition of (**a**) chromium (Cr), (**b**) iron (Fe), and (**c**) sulfur (S) in the surface of soil samples before and after treatment with 0.5 CMC@MMT-FeS and FeSO_4_ reagent.

The chemical composition of Cr, Fe, and S in the soil-sample surfaces, before and after 5% 0.5 CMC@MMT-FeS and 2.5 mmol FeSO_4_ reagent treatment, was detected using XPS; this was to investigate the mechanism of Cr(VI) immobilization in soils. Figure 6a0 shows the XPS spectra of Cr2p on the soil surface, with three peaks in Cr(VI)-contaminated soils, located at 577.7, 580.0, and 586.5 eV, corresponding to Cr_2_O_3_, Cr(OH)_3_, and Cr(VI), respectively [37,66]. The percentages of Cr(III) and Cr(VI) in the contaminated soil were about 44 and 56%, respectively, of the chromium present. For the FeSO_4_-treated soil samples, as shown in Figure 6a1, the percentage of Cr(VI) on the soil surface decreased from 56 to 25.6%, and that of Cr(III) increased from 44 to 74.5%, of which Cr(III) in the Cr(III)-Fe(III) complex accounted for about 8.4%. For 0.5 CMC@MMT-FeS-treated soil samples, the Cr(VI) peak in Figure 6a2 shifted slightly from 586.5 to 589.9 eV. Following 0.5 CMC@MMT-FeS treatment, the percentage of Cr(VI) decreased from 56 to 2.1%, whereas that of Cr(III) increased from 44 to 98%. Moreover, the Cr(III)-Fe(III) complex accounted for approximately 70% of the Cr present in the contaminated soil, indicating that the resulting Cr(III) combined with iron to form a stable iron-bound chromium compound in soils. These results demonstrate that Cr(VI) was converted to insoluble Cr(III) precipitate and was immobilized in soil after remediation [37]. Besides, the form of Cr in the composite-treated soil changed from acid-soluble fraction to iron-bound fraction after remediation.

Figure 6b_0_ shows the Fe2p spectra from the untreated soil sample, with four peaks at 714.2, 717.6, 720.1, and 727.8 eV, corresponding to Fe_2_O_3_, Fe(OH)_3_, Fe_2_SiO_4_, and Fe_3_O_4_, respectively. For the FeSO_4_-treated soil samples (Figure 6b_1_), the percentage of Fe_2_O_3_/Fe(OH)_3_ in the soil also increased, from 43.5 to 60% of the iron in the soil, after remediation. The peak of Fe(OH)_3_ shifted slightly, from 717.6 to 718.8 eV, and its percentage contribution to the iron in the soil increased from 2.6 to 23.8%. For the 0.5 CMC@MMT-FeS-treated soil samples (Figure 6b_2_), the Fe_2_O_3_/Fe(OH)_3_-binding energy shifted from 714.2 and 717.6, to 713.6 and 716.1 eV, and the peak of Fe_2_SiO_4_ shifted slightly from 720.1 to 721.1 eV. The iron elements in the FeS from the remediation materials were mainly converted Fe_2_O_3_/Fe(OH)_3_, which accounted for 52% of the iron in soil. These results show that the Fe(II) from the CMC@MMT-FeS was oxidized and formed Fe(III), which is likely to form a stable Fe(III)–Cr(III) complex in the soil during the Cr(VI) reduction process.

Figure 6c shows the S on the soil-sample surfaces before and after treatment. The binding energy of S(VI) shifted slightly from 171.1 to 170 eV, and the percentage of S(VI) on the soil surface decreased from 57.3 to 43.8% of the S in the soil, in the 0.5 CMC@MMT-FeS treatment. S(IV) binding energy shifted slightly from 176.2 to 175.9 eV, and the contribution of S(IV) increased from 12.7 to 24.3% of the S in the soil. The proportions of disulfide and polysulfide were essentially unchanged. This clarifies that the S(-II) component of the composite is oxidized to form the S(IV) compound when CMC@MMT-FeS is added to the soil.

According to the soil-sample physicochemical properties and XPS analysis, the following reactions account for the process of Cr(VI) removal and immobilization by modified materials [5]:

(6) $$\mathrm{FeS}\;+{\;H}_{2}O\;\to {\;\mathrm{Fe}}^{2+}\;+{\;\mathrm{HS}}^{-}\;+{\;\mathrm{OH}}^{-}$$

(7) $$3{\mathrm{Fe}}^{2+}\;+{\;\mathrm{HCrO}}_{4}{}^{-}\;+{\;H}^{+}\;\to \;3{\mathrm{Fe}}^{3+}\;+{\;\mathrm{Cr}}^{3+}\;+\;4H_{2}O$$

(8) $${\mathrm{HS}}^{-}\;+\;2{\mathrm{HCrO}}_{4}{}^{-}\;+\;7H^{+}\;\to {\;\mathrm{SO}}_{3}{}^{2-}\;+\;2{\mathrm{Cr}}^{3+}\;+\;5H_{2}O$$

(9) $${\mathrm{Cr}}^{3+}\;+\;3{\mathrm{OH}}^{-}\;\to \;\mathrm{Cr}{\left(\mathrm{OH}\right)}_{3}$$

(10) $${\mathrm{xCr}}^{3+}\;+\;\left(1\;–\;x\right){\mathrm{Fe}}^{3+}\;+\;3H_{2}O\;\to \;({\mathrm{Cr}}_{X}{\mathrm{Fe}}_{1-X}){\left(\mathrm{OH}\right)}_{3}+\;3H^{+}$$

However, for the FeSO_4_ treatment, Fe(II) was the only electron donor in the remediated soil system, and thus Cr(VI) reduction was mainly achieved by two processes: Formulas (7) and (10).

### 3.6. The Effect of Cr(VI) Remediation Evaluated Using the V. faba Micronucleus Method

The micronucleus test is used to detect the number of micronuclei in *V. faba* root-tip meristem cells, which reflects the degree of damage to chromosomes in cells. The biotoxicity of Cr(VI) in the soil before and after 0.5 CMC@MMT-FeS remediation was determined using this method. The root inhibition rate (RIR) and PI were used to assess the effect of Cr(VI) remediation on *V. faba* (Table 1). In the CMC@MMT-FeS-remediation groups, the RIR decreased from 0.76 to 0.22 and the PI dropped from 0.68 to 0.20 with the increase in the composite–soil mass proportion from 1 to 10%. This indicates that the biotoxicity for *V. faba* in the Cr(VI)-contaminated soil was alleviated after composite remediation, which can also be seen from the elongation rate of *V. faba* root tips (Figure S3). The RIR and PI of the composite treatment groups were lower than those of the FeSO_4_ treatment groups, when Fe(II) was added at 2.5 and 5.0 mmol. The results indicate that the toxicity of CMC@MMT-FeS on *V. faba* was less than that of FeSO_4_ at the same Fe(II) conditions, and this can be ascribed to the higher immobilization efficiency of the composite than FeSO_4_.

### 3.7. The Effect of Cr(VI) Remediation Evaluated Using E. foetida

Superoxide dismutase (SOD) is believed to be a biological indicator of the active oxidative-stress response mediated by pollutants. Peroxidase (POD) is used as one of the physiological indexes to assess the environmental biotoxicity of pollution. The activities of these enzymes (Table 2) reflected the degree to which *E. foetida* was subjected to contamination stress: the SOD activity levels of *E. foetida* on days 1 and 3 were higher than those of the control group, indicating that Cr(VI) causes the earthworms’ biological stress. On days 7 and 14, all of the *E. foetida* individuals were dead in the highly contaminated soil (at the 1% composite–soil mass proportion, and 0.5 mmol FeSO_4_ treatment), whereas they survived in the other treatments. This indicates that the toxic stress caused by Cr(VI) on *E. foetida* was alleviated when enough of the composite was added. For treatment groups with different amount of composite, the comparison of SOD activity levels in 5 and 10% groups and 1 and 2% groups was not obvious. The *E. foetida* SOD activity level was higher at the 5% than at the 10% composite–soil mass proportion. This shows that the toxic stress experienced by *E. foetida* was not greatly reduced with the decrease of the Cr(VI) concentration in composite treated-soils. The *E. foetida* POD activity levels increased in the first three days, which indicates that the stress on *E. foetida* caused by Cr(VI) was enhanced by the addition of the composite. From 3 to 14 days, the POD values decreased, demonstrating that the stress on *E. foetida* was reduced. POD activity was higher at the 1% composite–soil mass proportion than for the other treatments (from days 1–3), mainly because the Cr(VI) concentration was higher in the soil; at 14 days, all of the *E. foetida* in the 1% composite–soil mass proportion treatment were dead. However, the POD activity level did not change distinctly with the amount of composite added. In summary, *E. foetida* experienced less toxic stress from the Cr(VI) contaminated soil after 5 or 10% added composite treatment.

## 4. Conclusions

The study describes the efficacy of a novel CMC@MMT-FeS composite, which was prepared to remediate Cr(VI)-contaminated soils. The Cr(VI)-removal capacity of CMC@MMT-FeS was enhanced by increasing the FeS-particle loading. The immobilization of Cr(VI) in contaminated soil occurred mainly in the first 15 days (a sharp decrease on the first day, followed by a gradual decrease); the content of Cr(VI) in soil tended to stabilize after 15 days. CMC@MMT-FeS performed better than the FeSO_4_ reagent when the same amount of Fe(II) was added to soil samples. The soil Cr(VI) concentration decreased by 86.3% after 30 days when 0.5 CMC@MMT-FeS was added to the contaminated soil at a composite–soil mass proportion of 10%: we suggest that this dose could meet the risk-intervention requirement value for second-class construction land. Further, the soil Cr fractions changed from AE to mainly OD and RS after composite remediation, but predominantly to OD. The Fe(III)–Cr(III) complex was the primary product of the remediation process by CMC@MMT-FeS. Biotoxicity, assessed using *V. faba* and *E. foetida*, was greatly reduced by remediation. These findings demonstrate that CMC@MMT-FeS is a promising material for remediating Cr(VI)-contaminated soils.

## Figures and Tables

**Table 1:** Responses of *Vicia faba* to the soil Cr(VI) concentration before and after remediation.

Treatment	Cr(VI) Concentration (mg/L)	RIR	PI
CK	0	-	-
CS	40.75	0.79	-
CS + 1% 0.5 CMC@MMT-FeS	21.21	0.76	0.68
CS + 5% 0.5 CMC@MMT-FeS	1.61	0.24	0.31
CS + 10% 0.5 CMC@MMT-FeS	0.71	0.22	0.20
CS + 0.5 mmol FeSO_4_	22.82	0.72	0.75
CS + 2.5 mmol FeSO_4_	2.99	0.55	0.41
CS + 5.0 mmol FeSO_4_	0.98	0.60	0.54

RIR (root inhibition rate) = 1−L_remediation_/L_CK_; L_remediation_ and L_CK_: mean root-tip length in the remediation and CK groups, respectively. PI (pollution index) = MCN_remediation_/MCN_CK_; MCN_remediation_ and MCN_CK_: *V. faba* micronucleus rate in the remediation and CK groups, respectively. CK: Control check (background soil); CS: contaminated soil. Micronucleus rate (MN) = N_micronucleus_/N_dividing_; N_micronucleus_ and N_dividing_: numbers of micronucleated cells and dividing cells, respectively. (-) No value.

**Table 2:** Enzyme activity of *Eisenia foetida* in soil samples on different days.

Treatment	SOD Values (U/mg Protein)				POD Values (U/mg Protein)			
	1 d	3 d	7 d	14 d	1 d	3 d	7 d	14 d
CK	1.93	4.89	8.91	9.40	6.81	9.40	8.03	7.08
CS + 1% 0.5 CMC@MMT-FeS	6.86	7.28	3.83	*	7.75	10.82	8.19	*
CS + 5% 0.5 CMC@MMT-FeS	11.67	13.27	8.32	5.9	6.16	8.24	8.23	7.02
CS + 10% 0.5 CMC@MMT-FeS	9.82	12.42	7.45	3.65	6.04	8.46	8.11	7.04
CS + 0.5 mmol FeSO_4_	5.90	8.28	*	*	8.04	9.75	*	*
CS + 2.5 mmol FeSO_4_	8.96	7.96	7.6	6.30	6.37	9.24	8.11	7.15
CS + 5.0 mmol FeSO_4_	6.61	9.82	11.73	11.03	6.48	9.16	8.16	7.25

* indicates that *Eisenia foetida* in the samples were dead; CK: control check, CS: contaminated soil. SOD: superoxide dismutase. POD: peroxidase. 0.5 CMC@MMT-FeS: CMC@MMT-FeS containing 0.5 mmol/g FeS.

## Supplementary Materials

The following are available online at https://www.mdpi.com/1660-4601/17/17/6087/s1, **Section 1:** Pretreatment of montmorillonite (MMT). **Section 2:** Modified European Community Bureau of Reference (BCR) sequential extraction tests. **Section 3:** Pretreatment of *Vicia faba* seedlings for root micronucleus tests. **Section 4:** Preparation of the soil extract for the *Vicia faba* micronucleus test. **Section 5:** Removal of Cr(VI) by CMC@MMT-FeS in aqueous solution. **Table S1**: Physical and chemical properties of the contaminated soil samples. **Table S2**: Solutions used in the modified European Community Bureau of Reference (BCR) method. **Table S3**: Concentration of Cr(VI) in soil samples for the *Eisenia foetida* experiments. **Table S4**: Fitting parameters of the kinetic model of Cr(VI) removal in aqueous solution. **Figure S1:** Effect of FeS loading on Cr(VI) removal efficiency in aqueous solution. **Figure S2:** Total Cr in the contaminated soil before and after remediation. **Figure S3:** Elongation rate of *Vicia faba* root tips in different treatment groups.
