# Supplementary material for: Immobilization of Cr(VI) in Soil Using a Montmorillonite-Supported Carboxymethyl Cellulose-Stabilized Iron Sulfide Composite: Effectiveness and Biotoxicity Assessment
Source: Int J Environ Res Public Health. 2020 Aug 21;17(17):6087. doi: 10.3390/ijerph17176087 (PMC7503541; doi:10.3390/ijerph17176087)
Supplement: Supplementary file 1 [file ijerph-17-06087-s001.pdf]

Supplementary Materials

Immobilization of Cr(VI) in soil using a montmorillonite-supported carboxymethyl cellulose-stabilized iron sulfide composite: Effectiveness and biotoxicity assessment

Dading Zhang [1,†], Yanqiu Xu [1,†], Xiaofei Li [2], Zhenhai Liu [3], Lina Wang [1], Chaojun Lu [4], Xuwen He [1], Yan Ma [1,*], Dexun Zou [2,*]

[1] School of Chemical and Environmental Engineering, China University of Mining and Technology (Beijing), Beijing 100083, China; tmaczdd02@163.com(D.Z.) xuyq15871041327@163.com(Y.X.); m18850341814@163.com (L.W.); 108137@cumtb.edu.cn (X.H.); yanma@cumtb.edu.cn (Y.M.)
[2] College of Chemical Engineering, Beijing University of Chemical Technology, Beijing 100029, China; lixiaofei203@126.com (X.L.); zoudx@mail.buct.edu.cn (D.Z.)
[3] College of Environmental Science and Engineering, Nankai University, Tianjin 300350, China; hiram0823@163.com (Z.L)
[4] Chinese Research Academy of Environmental Sciences, Beijing 100012, China; lucj@craes.org.cn (C.L)

* Correspondence: yanma@cumtb.edu.cn (Y.M); zoudx@mail.buct.edu.cn (D.Z.).; Tel.: +86 158-1012-8622 (Y.M.); +86-159-1066-5628 (D.Z.)

† Co-first authors.

Contents

Section 1. Pretreatment of montmorillonite (MMT).

Section 2. Modified European Community Bureau of Reference (BCR) sequential extraction tests.

Section 3. Pretreatment of *Vicia faba* seedlings for root micronucleus tests.

Section 4. Preparation of the soil extract for the *Vicia faba* micronucleus test.

Section 5. Removal of Cr(VI) by CMC@MMT-FeS in aqueous solution.

Table S1. Physical and chemical properties of the contaminated soil samples.

Table S2. Solutions used in the modified European Community Bureau of Reference (BCR) method.

Table S3. Concentration of Cr(VI) in soil samples for the *Eisenia foetida* experiments.

Table S4. Fitting parameters of the kinetic model of Cr(VI) removal in aqueous solution.

Figure S1. Effect of FeS loading on Cr(VI) removal efficiency in aqueous solution.

Figure S2 Total Cr in the contaminated soil before and after remediation.

Figure S3 Elongation rate of *Vicia faba* root tips in different treatment groups.

Section 1. Pretreatment of Montmorillonite (MMT)

To remove the residual carbon and metal ions from the crystal backbone, 9.55 g MMT was activated using 0.06% HCl solution (solid:liquid, 1:20) combined with mechanical stirring for 20 min and ultrasonic treatment for 120 min, to improve the purity of the MMT. The purified MMT was filtered to remove the supernatant, and washed with deionized water, until there was no further precipitant when $AgNO_3$ was added into the deionized water that had been used for washing. The purified MMT was then transferred into a 2-L anaerobic Erlenmeyer flask, and high purity nitrogen gas was added. This purified MMT was used to prepare the CMC@MMT-FeS composite.

Section 2. Modified European Community Bureau of Reference (BCR) sequential extraction tests

Briefly, step 1 involved adding 1 g of the soil sample and 40 mL 0.11 mol/L acetic acid (see Table S2 for a list of solutions) to a 100-mL centrifuge tube, followed by shaking for 16 h at 22 ± 5°C for extraction. The supernatant liquid was maintained at 4°C, and the metal concentration was then analyzed. In step 2, 40 mL of freshly prepared 0.5 M hydroxylamine hydrochloride was added to the residue from the first step. The reaction system was shaken for 16 h at 22 ± 5°C for extraction. The supernatant liquids were maintained at 4°C for subsequent analysis. In step 3, 10 mL of 30% hydrogen peroxide (v/v) was added to the residue from the second step, and the reaction system was digested at room temperature for 1 h. The digestion was performed in a water bath at 85 ± 2°C until the solution in the tube was less than 3 mL. Then, 10 mL of 30% hydrogen peroxide was again added, and this process was repeated until the solution in the tube was less than 1 mL. Thereafter, 50 mL of 1 M ammonium acetate was added to the tube, followed by shaking for 16 h at 22 ± 5°C. The supernatant liquids were maintained at 4°C. Finally, the remaining solid residue was digested with aqua regia, following the ISO 11466 protocol.

Section 3. Pretreatment of *Vicia faba* seedlings for root micronucleus tests

The disinfected seeds were soaked in pure water at 25°C for 24 h to allow the shell to peel off and were then placed between two wet cotton pieces for pre-germination. The primary roots were about 3–5 cm long after 3–5 days, and they were suspended in Hoagland's solution after their tips were cut off. The length of the secondary roots (1–2 cm) was suitable for the micronucleus test. The secondary roots were treated with soil extracts for 30 h.

Section 4. Preparation of the soil extract for the *Vicia faba* micronucleus test

Pure water was added to the soil at a solid–liquid ratio of 1:10; this was mixed, then oscillated at room temperature (23 ± 2°C) for 24 h. The mixture was refrigerated at 4°C for 24 h, and the supernatant was skimmed off. NaOH solution or dilute nitric acid was used to adjust the pH of the solution to 7.0 ± 0.5. The supernatant was filtered through a 0.45-μm membrane and the Cr(VI) concentration was determined.

Section 5. Removal of Cr(VI) by CMC@MMT-FeS in aqueous solution

All batch experiments were conducted in 100-mL Erlenmeyer flasks capped with Parafilm M. Using 0.01 M $NaNO_3$ as the background solution, a stock solution of Cr(VI) ions (500 mg/L, K_2CrO_4) was prepared and diluted appropriately, according to the required initial concentrations. Appropriate volumes of the stock solution were used to prepare experimental solutions with Cr(VI) ion concentrations of 5, 10, 20, 40, 50, 60, 90, 120, and 200 mg/L. Then, 100 mg of the composite (at 0.2, 0.5, and 1.0 mmol/g FeS, hereafter referred to as 0.2 CMC@MMT-FeS, 0.5 CMC@MMT-FeS, and 1.0

CMC@MMT-FeS, respectively) was placed in an Erlenmeyer flask, and 25 mL of the experimental solution (initial pH: 7.2 ± 0.5) at one of the initial concentrations was added, at a solid–liquid ratio of 1:250. The Erlenmeyer flasks were placed in a shaker at 120 rpm at 25°C for 6–8 h, after which the solutions were filtered through a 0.45-μm microfilter. A kinetic experiment of the composite's ability to remove Cr(VI) from solution by modified composite was conducted: 1 g of the 0.5 CMC@MMT-FeS was added to 1-L beaker containing 250 mL 50 mg/L Cr(VI) solution (initial pH: 7.2 ± 0.5), while stirring mechanically. Samples were taken at 1, 5, 10, 15, 20, 30, and 60 min, respectively. The Cr(VI) concentration of the sample was determined by spectrophotometry at 540 nm. The removal efficiency of Cr(VI) in solution was calculated as follows:

$$\mathrm{Cr(VI)\ removal\ efficiency\ (\%)} = (1 - \mathrm{C_t}/\mathrm{C_0}) \times 100$$

where C_0 and C_t are the concentrations (mg/L) of Cr(VI) in the solution initially and at time t, respectively. All experiments were conducted in duplicate

Table S1. Physical and chemical properties of the contaminated soil samples.

Soil property	Redox potential (mV)	Organic matter (g/kg)	Cation exchange capacity (cmol/kg)	pH	Cr(VI) content (mg/kg)	Total Cr content (mg/kg)
Test value	227	15.6	26.0	8.16	424.59	718.05

Table S2. Solutions used in the modified European Community Bureau of Reference (BCR) method.

Step	Solution
1	0.11 M acetic acid
2	0.5 M hydroxylamine hydrochloride
3	30% hydrogen peroxide,<br>1 M ammonium acetate
4	Aqua regia

Table S3. Concentrations of Cr(VI) in soil samples for the *Eisenia foetida* experiments.

Soil samples	Cr(VI) concentration (mg/L)	Dilution ratio	Cr(VI) concentration of diluted soil (mg/L)
CK	24	--	--
CS + 1% 0.5 CMC@MMT-FeS	268.20	2	146.35
CS + 5% 0.5 CMC@MMT-FeS	84.59	2	54.50
CS + 10% 0.5 CMC@MMT-FeS	58.36	3.5	34.41
CS + 0.5 mmol $FeSO_4$	342.44	2	183.50
CS + 2.5 mmol $FeSO_4$	219.67	3.7	76.34
CS + 5.0 mmol $FeSO_4$	75.41	2	50.02

CK: control check; CS: contaminated soil; -- No value; 0.5 CMC@MMT-FeS: CMC@MMT-FeS containing 0.5 mmol/g FeS.

Table S4. Fitting parameters of the kinetic model of Cr(VI) removal in aqueous solution.

Fitting model	k_{obs} (min^{-1})	R^2
Pseudo-second-order kinetic model	0.063	0.9931
Pseudo-second-order kinetic model	0.173	0.9999

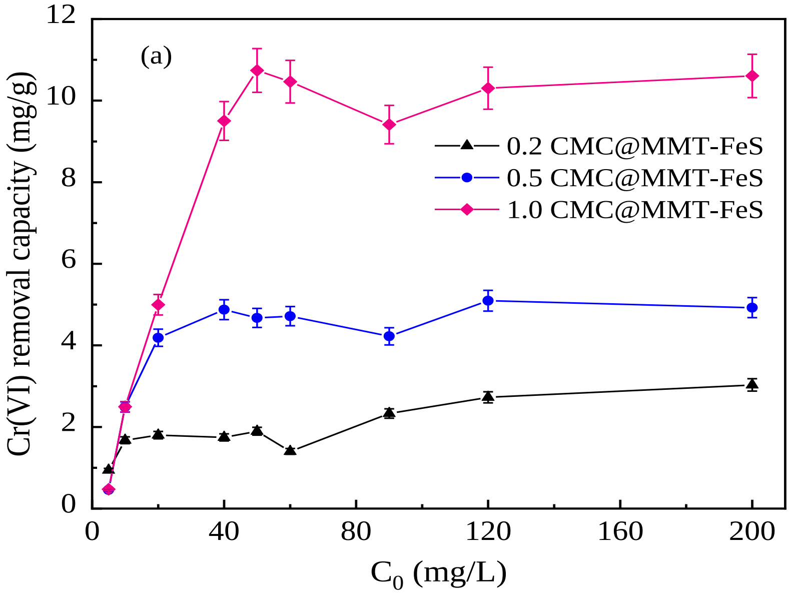

Figure S1 Effect of FeS loading on Cr(VI) removal efficiency in aqueous solution. The initial pH was 7.2; the temperature was 25°C; the solid:liquid ratio was 1:250. C_0: initial Cr(VI) concentration; 0.2, 0.5, and 1.0 CMC@MMT-FeS: CMC@MMT-FeS containing 0.2, 0.5, or 1.0 mmol/g FeS.

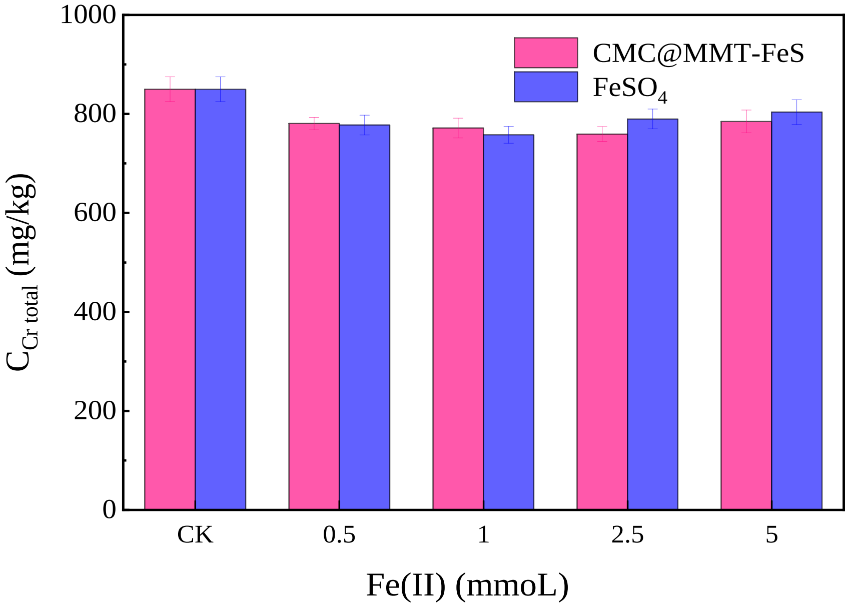

Figure S2 Total Cr in the contaminated soil before and after remediation. Addition amount of 0.5 CMC@MMT-FeS (i.e. composite containing FeS at 0.5 mmol/g) :1%, 2%, 5% and 10%. CK: control check.

The determination of total Cr in soil was based on Soil and sediment–Determination of aqua regia extracts of 12 metal elements–Inductively coupled plasma spectrometry (HJ803–2016, China). Briefly, the total Cr in soils was extracted with aqua regia (HCl/HNO_3 solution, 3:1) using an electric heating plate and the concentration in the extracts was measured using ICP–MS.

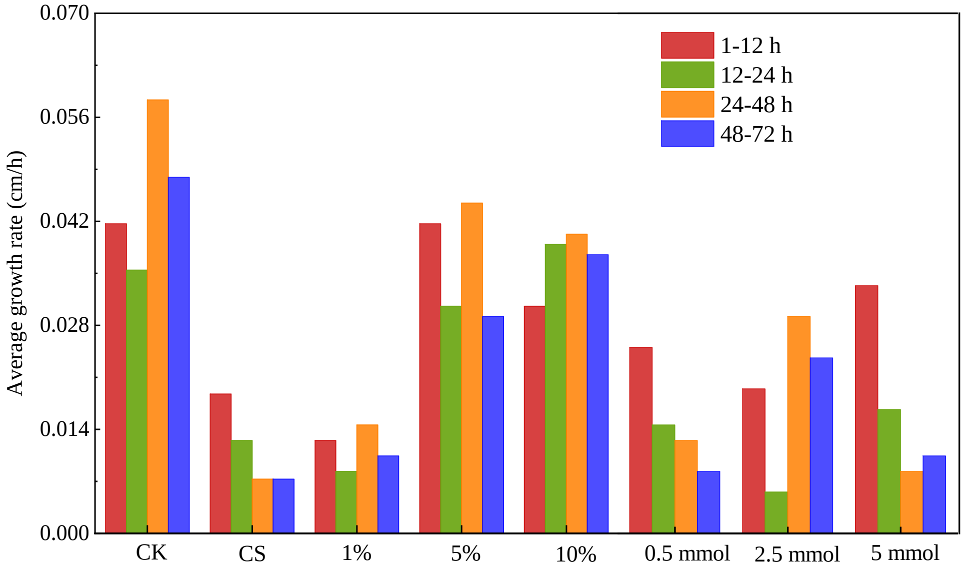

Figure S3 Elongation rate of *Vicia faba* root tips in different treatment groups. CK: control check, background soil; CS: Cr(VI)-contaminated soil; 1%, 5%, and 10%: soils after 1, 5, and 10% 0.5 CMC@MMT-FeS remediation; 0.5, 2.5, and 5 mmol: soils after 0.5, 2.5, and 5 mmol $FeSO_4$ remediation; 0.5 CMC@MMT-FeS: CMC@MMT-FeS containing 0.5 mmol/g FeS.
